# In Vitro Reassortment between Endemic H1N2 and 2009 H1N1 Pandemic Swine Influenza Viruses Generates Attenuated Viruses

**DOI:** 10.1371/journal.pone.0039177

**Published:** 2012-06-13

**Authors:** Ben M. Hause, Emily A. Collin, Zhiguang Ran, Laihua Zhu, Richard J. Webby, Randy R. Simonson, Feng Li

**Affiliations:** 1 Newport Laboratories, Worthington, Minnesota, United States of America; 2 Department of Biology and Microbiology, South Dakota State University, Brookings, South Dakota, United States of America; 3 Department of Veterinary and Biomedical Sciences, South Dakota State University, Brookings, South Dakota, United States of America; 4 Department of Infectious Diseases, St. Jude Children's Research Hospital, Memphis, Tennessee, United States of America; Centers for Disease Control and Prevention, United States of America

## Abstract

The pandemic H1N1 (pH1N1) influenza virus was first reported in humans in the spring of 2009 and soon thereafter was identified in numerous species, including swine. Reassortant viruses, presumably arising from the co-infection of pH1N1 and endemic swine influenza virus (SIV), were subsequently identified from diagnostic samples collected from swine. In this study, co-infection of swine testicle (ST) cells with swine-derived endemic H1N2 (MN745) and pH1N1 (MN432) yielded two reassortant H1N2 viruses (R1 and R2), both possessing a matrix gene derived from pH1N1. In ST cells, the reassortant viruses had growth kinetics similar to the parental H1N2 virus and reached titers approximately 2 log_10_ TCID_50_/mL higher than the pH1N1 virus, while in A549 cells these viruses had similar growth kinetics. Intranasal challenge of pigs with H1N2, pH1N1, R1 or R2 found that all viruses were capable of infecting and transmitting between direct contact pigs as measured by real time reverse transcription PCR of nasal swabs. Lung samples were also PCR-positive for all challenge groups and influenza-associated microscopic lesions were detected by histology. Interestingly, infectious virus was detected in lung samples for pigs challenged with the parental H1N2 and pH1N1 at levels significantly higher than either reassortant virus despite similar levels of viral RNA. Results of our experiment suggested that the reassortant viruses generated through *in vitro* cell culture system were attenuated without gaining any selective growth advantage in pigs over the parental lineages. Thus, reassortant influenza viruses described in this study may provide a good system to study genetic basis of the attenuation and its mechanism.

## Introduction

The pandemic H1N1 influenza A virus (pH1N1), first isolated from humans in 2009, quickly spread to numerous other species, including swine, cats and ferrets [1]–[4]. Genetic analysis of 1,516 swine influenza viruses (SIVs) isolated in 2009–2010 identified 41 viruses related to pH1N1, indicating that the pH1N1 had become established in North American swine herds [5]. Subsequently, co-infection of endemic SIV and pH1N1 in swine generated numerous reassortant viruses. The first reassortant SIV was identified in Hong Kong in 2010 and it contained a pH1N1-derived neuraminidase (NA) gene, a Eurasian-lineage hemagglutinin (HA) gene, and six internal genes derived from a conserved combination of avian, human and swine genes, which has been known as the triple reassortant internal gene cassette (TRIG) [6]. More recently, a H1N2 reassortant was isolated in the United Kingdom that contained HA and NA genes derived from endemic SIV and the six internal genes from pH1N1 [7]. In Italy, a H1N2 reassortant virus was isolated with all gene segments derived from pH1N1 except NA [8]. Similarly, a H1N1 reassortant virus was also isolated in Germany with the same pattern of recombination [9]. A swine H3N2 and pandemic H1N1 reassortant has also been reported in Canadian mink and pigs [10].

As pH1N1 viruses have shown a propensity for reassortment *in vivo*, there is a concern that viruses with increased pathogenicity may arise. Supporting this possibility were studies done by co-infection of matrix 2 (M2)-knockout versions of H5N1 and pH1N1 viruses in a Madin-Darby canine kidney (MDCK) cell line expressing M2. These studies yielded 33 different viral genotypes. Among the 59 clones analyzed, two exhibited improved growth kinetics as compared to the parental strains [11]. Similarly, co-infection of differentiated human airway cells with both seasonal and pH1N1 viruses generated a reassortant virus with all gene segments derived from pH1N1 except HA and this virus displayed enhanced growth kinetics in cell culture and increased virulence in ferrets [12]. Using reverse genetics, a reassortant virus with seven genes derived from the pH1N1 virus and the NA from a seasonal H3N2 influenza virus grew to higher titers in cell culture and caused more severe lung pathology in ferrets [13]. Similar work generated pH1N1 and human seasonal influenza reassortant viruses and observed enhanced growth kinetics of these viruses *in vitro* [14]. It is evident that pH1N1 readily reassorts with other co-circulating viruses and that these reassortant viruses have the potential for increased transmission and virulence. More recently, two reassortant swine-origin H3N2 viruses were isolated from children in Indiana and Pennsylvania [15]. These viruses were similar to endemic H3N2 SIV except that they possessed a Eurasian-lineage matrix gene derived from pH1N1.

In North America, nine reassortant SIVs representing seven distinct genotypes were characterized [16]. These genotypes contained various combinations of pH1N1 and endemic SIV-derived genes; however, nearly all reassortants contained a Eurasian-lineage matrix (M) from pH1N1 and endemic NA genes that is suggestive of increased viral fitness of viruses possessing these features. Despite the repeated isolation of such reassortant viruses, the impact of pH1N1 M and endemic NA on the replication, transmission, and pathogenesis of endemic SIV has not been fully investigated. The objective of this study was to generate reassortant viruses *in vitro* that reflect those seen in the field and characterize them in detail for growth kinetics in cell culture as well as for pathogenicity and transmissibility in swine.

## Materials and Methods

### Ethics Statement

Swine challenge studies were performed at South Dakota State University and were approved by the Institutional Animal Care and Use Committee (approval number 11-051A) and were performed under biosafety level 2+ conditions.

### Cells, viruses, and growth kinetics

Swine testicle (ST) cells (ATCC) were grown in DMEM containing 5% fetal bovine serum at 37°C with 5% CO_2_. A/swine/Minnesota/0745/2010 H1N2 (MN745) and A/swine/Minnesota/0432/2010 H1N1 (MN432) were isolated from routine diagnostic specimens submitted to Newport Laboratories (Worthington, Minnesota). Viral isolation was performed on ST cells from nasal swabs collected from pigs displaying influenza-like illness. Full genome sequencing was performed at St. Jude Children's Research Hospital. For simplicity and clarity, H1N2 for MN745 and pH1N1 for MN432 are used throughout the manuscript. Viral growth studies were performed on a monolayer of ST or A549 cells (ATCC) using an inoculum of 1.0 TCID_50_/mL (tissue culture infectious dose 50) in triplicate. Samples were removed at 0, 24, 48, 72 and 96 hours and infectious viruses were titrated on the same cell line and the titers determined by the method of Spearman-Karber.

### Generation of reassortant viruses

T25 flasks containing a monolayer of ST cells were infected with either pH1N1 or H1N2 at a multiplicity of infection (MOI) of 0.1 or co-infected with both pH1N1 and H1N2 with various combinations of MOI's ranging from 0.1∶0.1, 1.0∶0.1 or 0.1∶1.0. 1 ml of inoculum(s) was added to each flask and the virus was adsorbed for 1 hour and then removed and 20 mL of DMEM was added back. Flasks were incubated at 37°C with 5% CO2 for 3–4 days and then 0.1 mL of fluid was passed to a T25 flask containing a confluent monolayer of ST's. This procedure was repeated for a total of 10 passages. A sample was collected from each flask after each passage and analyzed for the presence of H1N2-M and pH1N1-M and NA genes by real time-RT-PCR (rt-RT-PCR). Cell culture harvests of passage x+10 were titrated on ST cells and 10 plaques from each co-infection flask and one plaque from each parent virus flask were expanded. Plaques were analyzed by rt-RT-PCR to determine M and NA lineages and identify reassortant viruses. Two reassortant viruses, designated R1 and R2, were identified that contain pH1N1-derived M and H1N2-derived NA and these two viruses were used in this study.

### Genetic Analysis

RNA was extracted with the MagMAX-96 viral RNA isolation kit per manufacturer's instructions (Life Technologies). rt-RT-PCR was performed using QIAGEN QuantiTect RT-PCR (QIAGEN) and primers and probes designed to detect and differentiate the North American lineage M gene and pH1N1-derived Eurasian M gene [17]. For specific detection of the pH1N1 NA gene, primers and probes were designed targeting sequences unique to Eurasian-lineage NA. The pH1N1-NA specific PCR used primers NA Forward: 5′-TCTCCCTATCCAAACACCATTG-3′ and NA Reverse: 5′-AGACAATCCACGCCCTAATG-3′and NA probe: 5′- AGACGATACTGGACCACAACTGCC-3′ using Cy5 as the fluorophore. pH1N1-NA detection was multiplexed with the differential M gene primers and probes. For rt-RT-PCR, negative samples were assigned a value of 37.1, which corresponds to detection limit of the method. Viruses were sent to St. Jude Children's Research Hospital's Hartwell Sequencing Center for determination of full-genome sequence. Sequences for the parental viruses MN432 (pH1N1) and MN745 (H1N2) were submitted to GenBank with accession numbers JQ023759–JQ023774.

### Pathogenicity and transmission in swine

Plaque purified pH1N1, H1N2 and reassortant viruses R1 and R2 were expanded by one additional passage on ST cells and cell culture fluids were titrated on ST cells. Thirty-six 3-week-old pigs were obtained from a commercial high-health herd and their SIV-negative status at day 0 was confirmed using a commercial ELISA (FlockChek Avian Influenza MultiS-Screen Antibody Test Kit; Idexx Laboratories Inc, Westbrook, Maine). Nasal swabs were also negative for SIV by rt-RT-PCR prior to study commencement. Pigs were divided into five groups of four pigs housed in separate rooms. On day 0, pigs were challenged intranasally with 1 mL of 1×10^6^ TCID_50_/mL virus in each nare with either pH1N1, H1N2, R1 or R2 delivered by a syringe. Control pigs were mock challenged with 1 mL DMEM in each nare. On day one, four naïve pigs were added to each challenge group. Pigs were observed daily for clinical signs of influenza and their temperatures recorded. Nasal swabs were collected daily to assess viral shedding. Viral titers in nasal swabs were quantified by rt-RT-PCR. Pigs were euthanized by a lethal dose of pentobarbitol on day five post-challenge and lungs were harvested.

### Histopathology

Samples of lungs collected from infected pigs on day five post-challenge were fixed in 10% buffered formalin and submitted to the Iowa State University Veterinary Diagnostic Laboratory for histopathology and immunohistochemistry for SIV.

### Statistical Analysis

The Student's t-test was used to determine statistical significance of titers *in vitro* as well as for Ct values in nasal swabs and infectious viral titers in lung samples using a probability value of 0.05 to indicate significance using the JMP software program (SAS, Cary, NC)

## Results

### Generation of reassortant viruses *in vitro*

**Figure 1:**
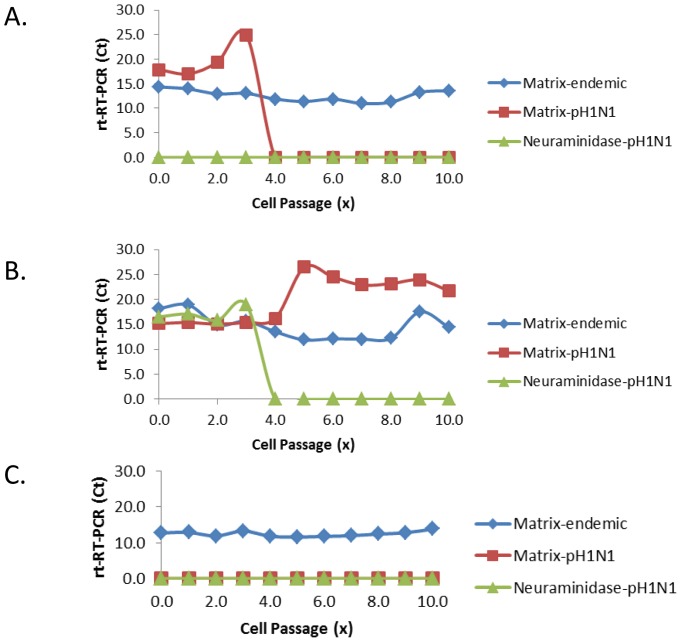
Flasks of ST cells were coinfected with H1N2 and pH1N1 with multiplicities of infection of 1∶1 (A), 0.1∶1.0 (B), and 1.0∶0.1 (C) of H1N2/pH1N1, respectively. The presence of the matrix genes derived from either H1N2 or pH1N1 as well as the neuraminidase derived from pH1N1 were quantified by rt-RT-PCR using gene specific primers and probes.

Routine characterization of SIV isolated from diagnostic samples identified MN745 as a H1N2 virus with homology to the δ-subcluster and MN432 as H1N1 with homology to pH1N1 based upon HA, M and NA gene sequencing. MN432 and MN745 were selected randomly as representatives of swine pH1N1 and endemic TRIG SIV, respectively. The percentage nucleotide identity for each segment between these two viruses is 94.0% for PB2, 94.2% for PB1, 94.1% for PB1, 76.0% for HA, 94.7% for NP, 53.8% for NA, 87.6% for M, and 94.2% for NS, respectively. rt-RT-PCR analysis of ST cell culture harvests after each passage indicated that flasks coinfected with viruses at MOI ratios of 1.0∶1.0 (Flask A) or 1.0∶0.1 H1N2∶pH1N1 (Flask C) only contained the endemic M gene from H1N2 (MN745) (Figure 1A, 1C). Additionally, the pH1N1-M and NA genes from pH1N1 (MN432) could not be detected after passage 10. In contrast, the flask coinfected with viruses at a MOI ratio of 0.1∶1.0 H1N2∶pH1N1 (Flask B) contained a mixed population after 10 passages as evidenced by positive PCR values for both the endemic and pH1N1-M genes (Figure 1B). In conjunction with the absence of signal for pH1N1-NA after 10 passages, these data also suggested that at least one of the viral populations present was a reassortant virus.

Ten plaques derived from each flask after the tenth passage were analyzed by rt-RT-PCR for the presence of endemic M and pH1N1-M and NA genes and partial HA gene sequence. Consistent with PCR data from cell culture harvests, all plaques from the flasks inoculated with MOI ratios of 1.0∶1.0 or 1.0∶0.1 H1N2∶pH1N1 had genotypes consistent with H1N2 based on HA, NA and M gene analysis. Eight of ten plaques from the flask coinfected with viruses at a MOI ratio of 0.1∶1.0 H1N2∶pH1N1 had a H1N2 genotype. The remaining two plaques were identified as reassortant viruses because they contained a pH1N1-derived M gene, were negative by PCR for pH1N1-NA but positive for H1N2 NA, and had HA gene sequences identical to H1N2.

### Genetic Analysis

Full genome sequences were determined for H1N2 and pH1N1 from the initial cell passage and following plaque purification after 10 sequential cell culture passages. Reassortants R1 and R2 were also sequenced following plaque purification. Pairwise ClustalW alignment of nucleotide sequences allowed determination of parentage of each gene (Table 1). For R1, polymerase basic 1 (PB1), polymerase acidic (PA), nucleoprotein (NP), M and NS (non-structural) genes were all derived from the pH1N1. The other three genes PB2 (polymerase basic 2), HA and NA were derived from H1N2. For R2, only PB1, NP and M were derived from pH1N1, with the remaining genes originating from H1N2.

**Table 1:** The lineage of the eight segments of the reassortant viruses genomes were determined by complete genome sequencing of reassortant viruses R1 and R2.

Virus	PB2	PB1	PA	HA	NP	NA	M	NS
R1	H1N2	pH1N1	pH1N1	H1N2	pH1N1	H1N2	pH1N1	pH1N1
R2	H1N2	pH1N1	H1N2	H1N2	pH1N1	H1N2	pH1N1	H1N2

Full genome sequences identified synonymous mutations in seven and non-synonymous mutations in six of the eight genes following 10 passages in cell culture. Nucleotide and amino acid changes, as compared to the parental gene sequence in the inoculum, are shown in Table 2. The majority of mutations were identified only in a single virus with the exception of a R528Q mutation that was found in the HA protein of H1N2 and both reassortants R1 and R2. Additionally, interestingly, both reassortants also had a S224R mutation in the M1 protein within the M segment, which was absent in both parental viruses.

**Table 2:** Single nucleotide polymorphisms identified in pH1N1 and H1N2 following 10 passages in ST cells as compared to genome sequences prior to cell passaging^a^.

Virus	PB2	PB1	PA	HA	NP	NA	M	NS
pH1N1				G193T (A65S)				
H1N2	A15G			G1583A (R528Q)		G1333A (V445M)	A4G (S2G)	
							G576A (M192I)	
R1				G1583A (R528Q)			C672A (S224R)	G385T (V129L)
R2	G2044A (G682S)		G679T C1866A	G1583A (R528Q)	G13A (E5K)		C672A (S224R)	

For non-synonymous mutations, amino acid changes are shown in parentheses.

a For reassortant viruses R1 and R2, single nucleotide polymorphisms are reported based on changes observed from the parent segment at x+0.

### *In vitro* growth kinetic experiment

**Figure 2:**
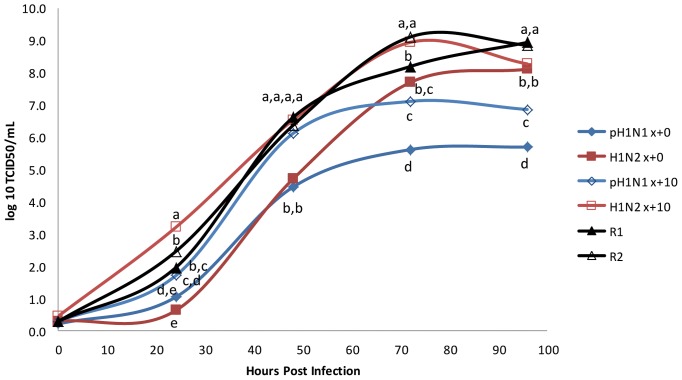
Multicycle growth curves for pH1N1 and H1N2, both before (x+0) and after 10 passes (x+10) in ST cells, as well as reassortant viruses R1 and R2. Data represent means of triplicate growth curves. Samples with different subscripts at a time point are statistically different (P<0.05).

Multiple cycle growth kinetics were performed on pH1N1 and H1N2, both prior to and following 10 sequential passages in ST cells, as well as for R1 and R2. In ST cells, R1 and R2 had maximal titers similar to parent H1N2 after 10 passages in cell culture and 2 log_10_ TCID_50_/mL higher than parent pH1N1 following ten cell culture passages (Figure 2). These differences were statistically significant (*P*<0.05, denoted by different subscripts). The maximal titer for both pH1N1 and H1N2 increased 1.4 and 0.8 log_10_ TCID_50_/mL, respectively following 10 cell culture passages. Statistical analysis of the differences of virus replication among different groups of infections here and elsewhere in the manuscript was performed by the Student's t-test coupled with the JMP software program. Statistically significant differences (P<0.05) for the level of virus replication at different time points among experimental groups were indicated by different subscripts (a, b, c, d, and e).

**Figure 3:**
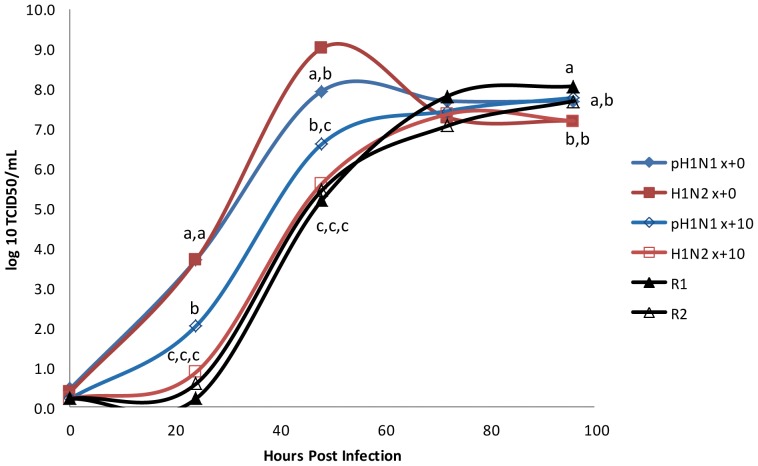
Multicycle growth curves for pH1N1 and H1N2, both before (x+0) and after 10 passes (x+10) in A549 cells, as well as reassortant viruses R1 and R2. Data represent means of triplicate growth curves.

Additionally, multiple cycle growth curves were performed using A549 cells. pH1N1 and H1N2, prior to passaging, reached maximal titers of 7.9 and 9.0 log_10_ TCID_50_/mL, respectively, at 48 hours (Figure 3). Sequential passage in ST cells decreased pH1N1 and H1N2 growth rates and titers as seen by a slower growth phenotype for viruses derived from x+10 in A549 cells. Reassortant viruses R1 and R2 had similar growth kinetics and maximal titers as compared to the two parents pH1N1 and H1N2 following 10 cell passages.

### Pig Studies

**Figure 4:**
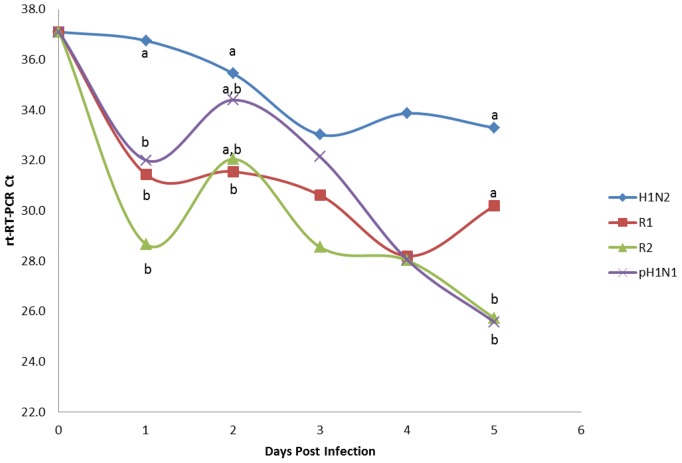
Four pigs were each challenged intranasally with 2 mL of 6.2 log10 TCID50/mL with H1N2, pH1N2, R1 or R2. Nasal swabs were collected daily and analyzed by rt-RT-PCR for virus shedding. RT-PCR data on day 0 reflect swabs collected prior to inoculation. Data represent the mean Ct value for the four nasal swabs collected at each time point. Samples with different subscripts at a time point are statistically different (P<0.05). Detection limit of rt-RT-PCR assay used in this study is approximately 1 infectious particle of SIV.

**Figure 5:**
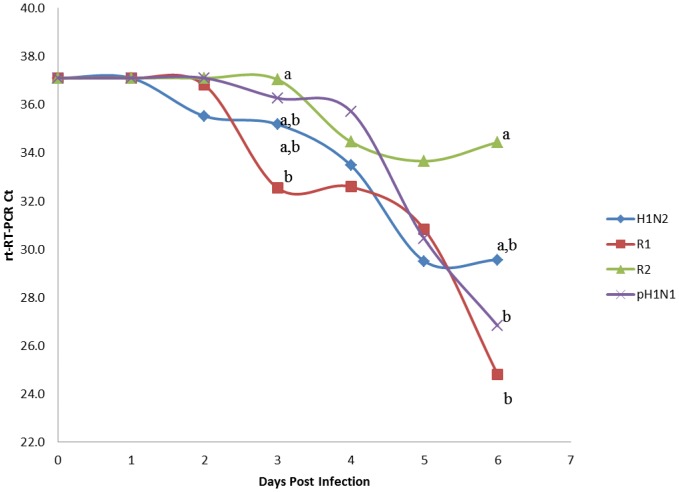
Four pigs were added to each of four rooms containing pigs challenged intranasally with 2 mL of 6.2 log10 TCID50/mL with H1N2, pH1N1, R1 or R2 at day one post infection. Nasal swabs were collected daily and analyzed by rt-RT-PCR for virus shedding. Data represent the mean Ct value for the four nasal swabs collected at each time point. Samples with different subscripts at a time point are statistically different (P<0.05).

**Figure 6:**
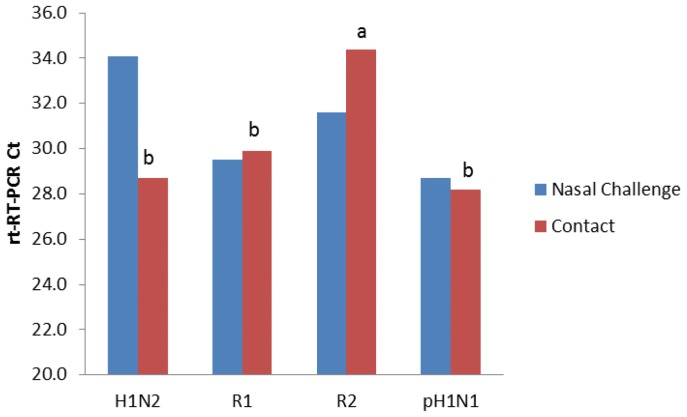
Lung samples were collected on day five post infection and analyzed for viral RNA by rt-RT-PCR. Data represent the mean Ct value for the four lung samples per treatment group. Samples with different subscripts within the same treatment group are statistically different (P<0.05).

**Figure 7:**
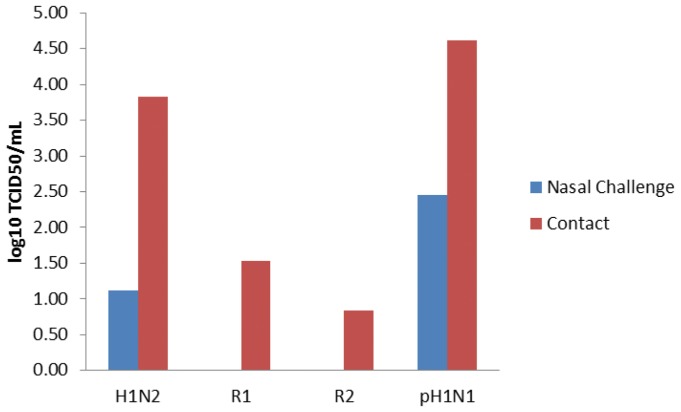
Lung samples were collected on day five post infection and analyzed for TCID50 by virus replication assay in ST cells. Data represent the mean log_10_ TCID_50_/mL for the four lung samples per treatment group. Samples with different subscripts within the same treatment group are statistically different (P<0.05).

SIV was detected in nasal swabs by rt-RT-PCR on day one post infection (p.i.) among all intranasally challenged pigs and was consistently detected until pigs were euthanized on day 5 p.i. (Figure 4). pH1N1 and R2 shedding was significantly higher (P<0.05) than H1N2 and R1 by 5 d.p.i. Similarly, virus was detected in naïve contact pigs beginning day one post exposure (day 2 post infection) continually until the pigs were euthanized (Figure 5). Interestingly, pH1N1 and R1 shed virus in contact exposure pigs at significantly higher levels than R2. Despite viral shedding, no clinical signs of influenza infection or elevated temperatures were observed (data not shown). All four viruses were capable of infecting and transmitting between pigs as determined by rt-RT-PCR. Lung samples collected on day 5 p.i. from directly challenged and day 5 p.e. contact pigs were all positive for virus by rt-RT-PCR with the exception of one pig directly infected with H1N2 and two contact pigs exposed to R2 (Figure 6). Titration of lung homogenates on ST cells only detected virus replication in two of the four pigs challenged with H1N2 (mean 0.5 log_10_ TCID_50_/mL) and three of the four pigs challenged with pH1N1 (mean = 1.8 log_10_ TCID_50_/mL, Figure 7). In contrast, all pigs challenged with R1 and R2 were negative for the presence of infectious viruses as measured in ST cells. Further analysis of lung homogenates from contact exposure pigs showed that these pigs were all positive for SIV with the exception of 1 pig exposed to R2. However, viral titers were significantly lower for reassortant viruses R1 and R2 (1.3 and 0.7 log_10_ TCID_50_/mL, respectively) than parental H1N2 and pH1N1 (3.6 and 3.4 log_10_ TCID_50_/mL, respectively). Histopathology of nasal challenge pigs found necrotizing bronchiolitis ranging from bronchiolar epithelial attenuation to necrosis. There were no discernible differences in microscopic lesions that could be attributed to different viruses. In addition, no obvious macroscopic lung lesions were observed in infected pigs. Immunohistochemistry (IHC) was positive for SIV in two of the four pigs intranasally challenged with H1N2 and three of the four pigs challenged with pH1N1, R1 and R2. Contact exposure pigs also contained lesions consistent with influenza infection, namely disorganization and sloughing of epithelial cells lining the airways, which were occasionally lightly cuffed with infiltrating lymphocytes. Approximately half of the pigs exposed to H1N2, R1 and pH1N1 were IHC positive for SIV. All pigs exposed to R2 were IHC negative for SIV. No lesions were detected in mock-challenged pigs and were IHC negative for SIV. Similarly, all nasal swabs from mock-challenged pigs were negative by rt-RT-PCR and no virus was detected in lung samples by rt-RT-PCR or titration in cells (data not shown).

## Discussion

Shortly after the identification of pH1N1 in humans in North America, the virus spread rapidly worldwide, becoming the first pandemic of the 21^st^ century. Soon thereafter, pH1N1 was identified in numerous species, including swine. The pH1N1 contains two genes (NA and M) of Eurasian-lineage viruses, which have not been seen before in North America. A primary concern is that addition of new genetic material to the already diverse field strains of SIV circulating in North American pigs could result in reassortant viruses with increased pathogenicity and transmission to pigs or humans [18]. Reassortant viruses containing segments from endemic and pH1N1 have since been identified in numerous countries [1], [6]–[10], [18]. The genotype of HA and NA from endemic SIV and M from pH1N1 seems to be particularly favored and their wide-prevalence in swine populations might be due to increased viral fitness [16]. Similar to reassortant viruses characterized from field isolates, in this work co-infection of ST cells with swine H1N2 (MN745) and pH1N1 (MN432) isolates generated reassortant viruses (R1 and R2) that possessed genes for HA and NA from the endemic strain and a pH1N1-derived M gene. In both ST and A549 cells, R1 and R2 had growth kinetics similar to H1N2 and in the case of ST cells R1 and R2 reached titers significantly higher than pH1N1. Interestingly, cell passage of pH1N1and H1N2 increased viral titers in ST cells while having a negative effect on the growth kinetics on A549 cells. Sequencing of viruses prior to and after sequential cell passage identified a number of mutations that likely contribute to these phenotypes. For pH1N1, only one amino acid change was identified after cell passage (HA A65S). This mutation is presumably responsible for the 2 log_10_ TCID_50_/mL increase in titer observed for this virus in ST cells and decreased titers and growth rate in A549 cells. A conserved mutation was also observed in the HA for H1N2 and both reassortant viruses (R528Q). As other mutations were also present in these viruses, the relevance of this mutation requires additional research.

Following intranasal challenge of pigs, all viruses were detected in nasal swabs by rt-RT-PCR beginning one d.p.i., demonstrating that all viruses were capable of replicating *in vivo*. Viral shedding was significantly higher for pH1N1 and R2. Additionally, all viruses were able to transmit to contact pigs placed to the challenge rooms one day following intranasal challenge, with pH1N1 and R1 shed at significantly higher levels than R2. Presence of viral RNA was also demonstrated in lung samples from all pigs at day five post exposure. The only statistically significant difference in lung viral RNA levels were observed in contact pigs exposed to R2, where viral RNA levels were significantly less than other groups. This is in agreement with rt-RT-PCR data on nasal swabs where viral RNA levels in pigs exposed to R2 were significantly lower than other challenge groups. Titration of virus in lung homogenates identified infectious virus only for pH1N1 and H1N2 in intranasally challenged pigs. Infectious virus was seen in all four contact groups but titers were significantly higher for pH1N1 and H1N2. These results suggest that two reassortant viruses R1 and R2 are significantly attenuated *in vivo*, albeit exhibiting the parental capacity in replicating viral RNA genome. Similar results were seen when pigs were challenged with either a human or swine-origin pH1N1 virus where viral RNA for both the swine and human pH1N1 isolates were detected in lung samples at equivalent levels however the live virus titer was significantly higher in lungs of pigs infected with the human pH1N1 isolate [19].

The failure of the M segment from pH1N1 to offer R1 and R2 a replication advantage in pigs over the parental strains came as a surprise because field SIV isolates with the same genotype appear to have a better fitness and are dominant in North American pig farms. Additionally, Chou et al. reported that the M segment of the 2009 pH1N1 influenza virus promoted aerosol transmissibility [20]. In contrast, Ma et al. found that the combination of pH1N1-derived M and NA in context of the triple reassortant gene constellation was required for direct transmission in pigs [21]. Viruses containing only the pH1N1 M gene were attenuated both *in vitro* and *in vivo*. It is apparent that pH1N1 M gene functions are dependent on the context of other viral proteins. Despite its unclear mechanism, we believe that there are several possible explanations for this interesting observation. One possibility is that reassortment between endemic H1N2 and 2009 H1N1 pandemic swine influenza viruses created through *in vitro* cell culture system may not truly replicate the situation as observed in the field. The other possibility is that R1 and R2 viruses may transmit and replicate more efficiently in pigs through aerosol transmission route, which has not been addressed in this study. An alternative explanation is that S224R mutation in M1 protein within the M segment present only in R1 and R2 viruses might be responsible for the attenuation. Position 221 of M1 protein is located in the C-domain of M1 protein that has been viewed as a determinant for viral growth and virulence in mammalian hosts [22]. Interestingly, S224 is the first serine within the conserved triple serine motif (S224/S225/S226). PhosphoMotif finder revealed that the triple serine motif including S224 is likely a substrate for phosphorylation (data not shown). Given that M1 is evidently phosphorylated, we hypothesize that the S224R mutation abolishes phosphorylation at position 224 and as a result attenuates virus replication in pigs, which will be addressed in our future study [23]. As a starting point to elucidate the importance of the M1 S224 position, a S224R mutation was generated in a H1N1 WSN33 reverse genetics system and the derived virus was attenuated in MDCK cells approximately 10-fold in infectivity as compared to wild type virus (data not shown). This initial finding together with results presented in this manuscript warrant future investigation about the role of the M1 S224 position in influenza virus replication and pathogenesis. Additionally, a H222R mutation in the M gene was one of numerous mutations observed in a swine pH1N1 virus that also displayed an attenuated replication phenotype [19].

Histolopathological examination of lung samples found microscopic lesions and associated SIV for all intranasal challenge groups. Additionally, SIV was identified by IHC in lung lesions in contact-exposed pigs for pH1N1, H1N2, and R1. The failure to identify SIV or lung lesions in pigs exposed to R2 is consistent with significantly lower levels of R2 in nasal swabs and lung samples as determined by rt-RT-PCR. This result is surprising given that R2 was detected in high levels in nasal swabs and SIV associated with microscopic lung lesions were demonstrated in pigs intranasally challenged with R2. Unique mutations found in R2 included PB2 G682S and NP E5K. The role of these mutations in the apparently decreased transmissibility of SIV is of further interest. Alternatively, further mutations generated *in vivo* may be responsible for the observed phenotype. Mutations in the pH1N1 PB2 and NP genes have previously been shown to effect pathogenicity of pH1N1 viruses in mouse models [24], [25].

In summary, using *in vitro* cell culture system, we have generated two reassortant influenza viruses carrying the M segment from the human 2009 pandemic lineage and HA and NA genes from endemic swine influenza viruses. These two reassortant viruses replicated efficiently in MDCK and A495 cells but were attenuated in pigs. In addition to have practical value for attenuated live vaccine development against swine influenza, reassortant viruses reported in this study may provide a good system to explore the role of M and other potential segments in viral transmissibility and pathogenesis.

## References

[1] Howden KJ, Brockhoff EJ, Caya FD, McLeod LJ, Lavoie M (2009). An investigation into human pandemic influenza virus (H1N1) 2009 on an Alberta swine farm.. Can Vet J.

[2] Löhr CV, DeBess EE, Baker RJ, Hiett SL, Hoffman KA (2010). Pathology and viral antigen distribution of lethal pneumonia in domestic cats due to pandemic (H1N1) 2009 influenza A virus.. Vet Pathol.

[3] Nfon CK, Berhane Y, Hisanaga T, Zhang S, Handel K (2011). Characterization of H1N1 swine influenza viruses circulating in Canadian pigs in 2009.. J Virol.

[4] Swenson SL, Koster LG, Jenkins-Moore M, Killian ML, DeBess EE (2010). Natural cases of 2009 pandemic H1N1 influenza A virus in pet ferrets.. J Vet Diagn Invest.

[5] Nelson MI, Lemey P, Tan Y, Vincent A, Lam TT (2011). Spatial dynamics of human-origin H1 influenza A virus in North American swine.. PLoS Pathog.

[6] Vijaykrishna D, Poon LLM, Zhu HC, Ma SK, Li OTW (2010). Reassortment of pandemic H1N1/2009 influenza A virus in swine.. Science.

[7] Howard WA, Essen SC, Strugnell BW, Russell C, Barrass L (2011). Reassortant pandemic (H1N1) 2009 virus in pigs, United Kingdom.. Emerg Infect Dis.

[8] Moreno A, Trani LD, Faccini S, Vaccari G, Nigrelli D (2011). Novel H1N2 swine influenza reassortant strain in pigs derived from the pandemic H1N1/2009 virus.. Vet Microbiol.

[9] Starick E, Lange E, Fereidouni S, Bunzenthal C, Höveler R (2011). Reassorted pandemic (H1N1) 2009 influenza A virus discovered from pigs in Germany.. J Gen Virol.

[10] Tremblay D, Allard V, Doyon JF, Bellehumeur C, Spearman JG (2011). Emergence of a new swine H3N2 and pandemic (H1N1) 2009 influenza A virus reassortant in two Canadian animal populations, mink and swine.. J Clin Microbiol doi.

[11] Octaviani CP, Ozawa M, Yamada S, Goto H, Kawaoka Y (2010). High level of genetic compatibility between swine-origin H1N1 and highly pathogenic avian H5N1 influenza viruses.. J Virol.

[12] Ilyushina NA, Ducatez MF, Rehg JE, Marathe BM, Marjuki H (2010). Does pandemic A/H1N1 virus have the potential to become more pathogenic?. mBio.

[13] Schrauwen EJA, Herfst S, Chutinimitkul S, Bestebroer TM, Rimmelzwaan GF (2011). Possible increased pathogenicity of pandemic (H1N1) 2009 influenza virus upon reassortment. Emerg.. Infect Dis.

[14] Octaviani CP, Li C, Noda T, Kawaoka Y (2011). Reassortment between seasonal and swine-origin H1N1 influenza viruses generates viruses with enhanced growth capability in cell culture.. Virus Res.

[15] Centers for Disease Control and Prevention (2011). Swine-origin influenza A (H3N2) virus infection in two children-Indiana and Pennsylvania, July-August 2011.. MMWR Morb Mortal Wkly Rep.

[16] Ducatez MF, Hause B, Stigger-Rosser E, Darnell D, Corzo C (2011). Multiple reassortment between pandemic (H1N1) 2009 and endemic influenza viruses in pigs, United States. Emerg Infect Dis..

[17] Harmon K, Bower L, Kim WI, Pentella M, Yoon KJ (2010). A matrix gene-based multiplex real-time RT-PCR for detection and differentiation of 2009 pandemic H1N1 and other influenza A viruses in North America.. Influenza Other Respi Viruses.

[18] Zhu H, Zhou B, Fan X, Lam TT, Wang J (2011). Novel reassortment of Eurasian avian-like and pandemic/2009 influenza viruses in swine: infectious potential for humans.. J Virol.

[19] Weingartl HM, Berhane Y, Hisanaga T, Neufeld J, Kehler H (2010). Genetic and pathobiologic characterization of pandemic H1N1 2009 influenza viruses from a naturally infected swine herd.. J Virol.

[20] Chou Y, Albrecht RA, Pica N, Lowen AC, Richt JA (2011). The M segment of the 2009 new pandemic H1N1 influenza virus is critical for its high transmission efficiency in the guinea pig model.. J Virol.

[21] Ma W, Liu Q, Bawa B, Qiao C, Qi W (2012). The NA and M genes of the 2009 pandemic influenza H1N1 virus functionally cooperate to facilitate efficient replication and transmissibility in pigs.. J Gen Virol Doi.

[22] McCullers JA, Hoffmann E, Huber VC, Nickerson AD (2005). A single amino acid change in the C-terminal domain of the matrix protein M1 of influenza B virus confers mouse adaptation and virulence.. Virology.

[23] Gregoriades AT, Christie T, Markarian K (1984). The membrane (M1) protein of influenza virus occurs in two forms and is a phosphoprotein.. J Virol.

[24] Sakabe S, Ozawa M, Takano R, Iwastuki-Horimoto K, Kawaoka Y (2011). Mutations in PA, NP, and HA of a pandemic (H1N1) 2009 influenza virus contribute to its adaptation to mice.. Virus Res.

[25] Song M-S, Noriel PQ, Lee JH, Baek YH, Park KJ (2011). Virulence and genetic compatibility of polymerase reassortant viruses derived from the pandemic (H1N1) 2009 influenza virus and circulating influenza A viruses.. J Virol.

